# Genome Sequences of the *Mycobacterium tuberculosis* H37Rv-*ptkA* Deletion Mutant and Its Parental Strain

**DOI:** 10.1128/genomeA.01156-17

**Published:** 2017-11-02

**Authors:** Clement K. M. Tsui, Dennis Wong, Gagandeep Narula, Jennifer L. Gardy, William W. H. Hsiao, Yossef Av-Gay

**Affiliations:** a Division of Infectious Diseases, Faculty of Medicine, University of British Columbia, Vancouver, BC, Canada; b School of Population and Public Health, University of British Columbia, Vancouver, BC, Canada; c Communicable Disease Prevention and Control Services, British Columbia Centre for Disease Control, Vancouver, BC, Canada; d British Columbia Centre for Disease Control Public Health Laboratory, Vancouver, BC, Canada; e Department of Pathology and Laboratory Medicine, University of British Columbia, Vancouver, BC, Canada

## Abstract

*Mycobacterium tuberculosis*, the etiological agent of tuberculosis, is one of the most devastating infectious agents in the world. Here, we report the draft genome sequences of the *M. tuberculosis* protein tyrosine kinase (*ptkA*) deletion mutant and its parental strain H37Rv, which are used in genetic studies and for drug discovery.

## GENOME ANNOUNCEMENT

*Mycobacterium tuberculosis* is a great threat to humanity, and it kills about two million people annually (1). *M. tuberculosis* is able to subvert the killing machinery of macrophages, a key component of the human innate immune system, and replicate inside the macrophage in an organelle termed the phagosome (2). *M. tuberculosis* evades the host immune system and is protected from chemotherapies that fail to reach the phagosome (2). There is an urgent need to better understand *M. tuberculosis* pathogenesis, particularly the role of its signaling molecules, e.g., the protein tyrosine kinase (PtkA). Studies showed that PtkA phosphorylates protein tyrosine phosphatase A (PtpA) and enhances its phosphatase activity; PtpA plays a key role in *M. tuberculosis* pathogenesis, enabling the inhibition of host phagosome maturation processes (3, 4).

A deletion mutant was constructed according to the method reported by Bardarov et al. (5) to investigate the role of *ptkA* in *M. tuberculosis* pathogenesis. The mutant was derived from the parental strain H37Rv, which has been propagated in the Av-Gay laboratory. The parental strain is also being used in the intracellular drug-screening assay to identify lead compounds that are effective against *M. tuberculosis* within the human macrophage (6, 7). Since independent mutations could have accumulated in stock H37Rv cultures of different laboratories (8), determining the genome sequences of the *ptkA* deletion mutant and its parental H37Rv strain would allow the functional investigation of targeted gene knockouts and the characterization of single nucleotide variants (SNVs) that affect drug tolerance and metabolism.

The *M. tuberculosis ptkA* deletion mutant and the parental strain were plated on 7H10 agar plates and incubated at 37°C in humidified air (7). Genomic DNA was extracted using the lysozyme method (9). The paired-end (PE) DNA libraries were constructed with a Nextera XT DNA kit (Illumina, San Diego). The tagmented DNA was amplified by index primers and purified with AMPure XP beads to remove small library fragments. DNA libraries were normalized, pooled, and sequenced using the Illumina MiSeq platform at the British Columbia Centre for Disease Control Public Health Laboratory (BCCDC PHL) with 250-bp PE reads (MiSeq reagent kit v2).

The quality of the reads in fastq format was assessed by Fastqc (http://www.bioinformatics.babraham.ac.uk/projects/fastqc/). Reads were quality trimmed by Trim Galore (http://www.bioinformatics.babraham.ac.uk/projects/trim_galore/) and were assembled using SPAdes v.3.9.0 (10) with default settings. The statistics from the genome assemblies were summarized using QUAST (11). The assembled contigs were annotated using the Rapid Annotations using Subsystems Technology (RAST) server (version 2) (12).

Trimmed sequence reads were aligned to the reference genome sequence of H37Rv (GenBank accession number NC_000962.3) using BWA-mem (13). SNVs were called using GATK v.3 (14) and quality filtered using VCFtools (15) to ensure high confidence. SnpEff (16) was used to annotate the changes in SNVs observed between our laboratory stock strain and the standard reference H37Rv strain.

The genome sizes of the H37Rv-*ptkA* deletion mutant and its parent strain were 4,362,922 and 4,365,322 bp, with *N*_50_ values of 78,024 and 39,693 bp, and average depth coverages of 57× and 30×, respectively. The deletion of the *ptkA* gene was confirmed in the mutant genome assembly. Over 99% of the trimmed reads of the H37Rv parental strain were mapped to the reference genome sequence with polymorphisms at 27 sites (excluding SNVs in PPE and PE_PGRS genes).

### Accession number(s).

This whole-genome sequencing project has been deposited at DDBJ/ENA/GenBank under the accession numbers NSHG00000000 and NWUE00000000 under BioProject PRJNA400496.
